# The Taxonomic Diversity of Prokaryotic Communities from Permafrost Active Layers of the Chilean Andes

**DOI:** 10.3390/microorganisms14030613

**Published:** 2026-03-09

**Authors:** Viktória Faragó, Andrea K. Borsodi, Balázs Nagy

**Affiliations:** 1 Department of Microbiology, ELTE Eötvös Loránd University, H-1117 Budapest, Hungary; viktoriafarago27@gmail.com; 2 Doctoral School of Environmental Sciences, ELTE Eötvös Loránd University, H-1117 Budapest, Hungary; 3 Department of Physical Geography, ELTE Eötvös Loránd University, H-1117 Budapest, Hungary; balazs.nagy@ttk.elte.hu; 4 A Földgömb az Expedíciós Kutatásért Alapítvány, H-1142 Budapest, Hungary

**Keywords:** permafrost, active layer, extremophiles, microbial diversity, 16S rRNA gene-based amplicon sequencing, microbial ecology

## Abstract

The study of microorganisms inhabiting extreme environments offers a valuable opportunity to explore their potential ecological roles. This study aimed to reveal and compare the microbial taxonomic diversity of largely unexplored permafrost regions located in different climatic zones (dry and wet) in the Chilean Andes, separated by thousands of kilometers. Permafrost active layer samples were collected from the Ojos del Salado (Atacama Desert) and the Torres del Paine (Patagonia) from different sampling depths. Illumina 16S rRNA gene-based amplicon sequencing revealed that the Andean permafrost active layer provides diverse habitats for distinct microbial communities, with higher taxonomic diversity of Bacteria than Archaea. The wet Patagonian Andes samples showed higher diversity, with a greater abundance of *Chloroflexota* and *Bacteroidota*, while the dry Ojos del Salado samples were dominated by *Actinomycetota*, indicating desiccation stress. Archaea were classified as ammonia-oxidizing members of the *Thermoproteota* phylum. Beta-diversity analyses suggested that differences in environmental conditions (mainly available moisture) contributed more to community structure differentiation than geographical distances. Nevertheless, the effect of sampling depth on microbial diversity was insignificant.

## 1. Introduction

It is estimated that approximately 11% of Earth’s land surface is affected by permafrost on a global scale [1]. Permafrost is defined as ground that remains at or below 0 °C for at least two consecutive years [2]. Permafrost can have the capacity to store about 14.1 Pg (1 Pg = 10^15^ g) of soil organic C (SOC) in the top 3 m, which can be mobilized during permafrost thaw [3]. The upper 1–2 m of the permafrost, known as the active layer, is exposed to freeze-thaw cycles. Due to global warming, the active layer has thickened in numerous regions, leading to increased microbial activity and the release of greenhouse gases, such as methane and carbon dioxide [4]. Given the link between permafrost degradation and the exacerbation of global warming, there has been a surge in research interest in these regions.

Chile has a variety of extreme environments, including the Atacama Desert, which is the driest desert on Earth, as well as ice fields, cold lakes, and glaciers in Patagonia. Ojos del Salado (6893 m a.s.l.), located in the Dry Andes (27°06′33.84″ S, 68°32′29.63″ W) in the Atacama Desert, is the highest volcano on Earth and the highest mountain without substantial surface ice. Nevertheless, there may still be ground ice within the permafrost. Based on surveys of periglacial landforms and permafrost modeling, ground ice is expected to be above an elevation of 4000–5600 m in the Puna de Atacama [5,6,7]. The permanent snowline is around 7000 m as a result of severe drought and high irradiation [8]. Most precipitation occurs during the austral summer [9]. The Central Andes, particularly the Puna-Altiplano, has the highest surface solar radiation levels worldwide, with extreme values of approximately 310 W m^−2^ [10]. The area has been designated as a Mars analog research site due to conditions such as low average temperatures, large daily temperature variations, and frozen conditions at night, even during the summer [11].

Patagonia is characterized by a vast network of cold fjords, channels, and oligotrophic cold lakes that extend across southern Chile. The Torres del Paine National Park is located in southern Patagonia, with a total surface close to 181,000 ha, and it is in a transitional forest–steppe zone. The climate is a temperate cold rainy zone without a dry season. Rainfall measurements show significant variation between the eastern and western regions of the park, with annual precipitation ranging from 300 mm in the east to 1500 mm in the west. During the last glacial maximum, the park was entirely covered with ice, resulting in generally young soils [12]. The lithology is derived from volcanic deposits [13].

Microorganisms living in the permafrost have to tolerate sub-zero temperatures, limited water availability, and low-nutrient stress over extended periods of time [14]. Microorganisms capable of thriving in such inhospitable environments are classified as polyextremophiles, indicating their ability to endure multiple extreme conditions at the same time [15]. Despite the harsh environment, relatively high microbial diversity has been observed in permafrost. Certain microorganisms are active even at subzero temperatures. The bacterial diversity present within permafrost is typically higher in comparison to the diversity observed in archaea [16].

In permafrost, the presence of several bacterial phyla, including *Pseudomonadota*, *Chloroflexota*, *Acidobacteriota*, *Actinomycetota*, and *Bacteroidota* have been observed in the Qinghai–Tibet Plateau [17,18], Arctic permafrost [19], and the continuous permafrost region of northeastern China [20]. Previous research has revealed the presence of archaeal sequences, belonging to the *Euryarchaeota*, *Crenarchaeota*, and *Thaumarchaeota* [16]. Additionally, it is common to identify unclassified and novel taxa from extreme environments. The bacterial community of a seasonal high-altitude thaw pond at Ojos del Salado showed moisture-dependent shifts in the relative abundance of *Cyanobacteria* and *Actinomycetota*. The predominant taxa within the community included *Pseudomonadota*, *Bacteroidota*, *Acidobacteriota*, and *Verrucomicrobiota*, with multiple identified orders known for their ability to degrade a broad spectrum of organic compounds [21].

The aim of this study was to gain insight into the microbial taxonomic diversity of the hitherto almost undisturbed and unknown permafrost regions located in different climatic zones of the Andes in Chile. In addition to revealing and comparing the diversity of Bacteria, our research has also focused on uncovering the diversity of previously unknown communities of Archaea. We hypothesize that, although the samples were collected from the active layer of permafrost at both sampling sites, differences in microbial community composition are expected due to variation in geographic latitude and climatic conditions. We further propose that the depth of sampling influences microbial community structure.

## 2. Materials and Methods

### 2.1. Description of the Sampling Sites

**Figure 1:**
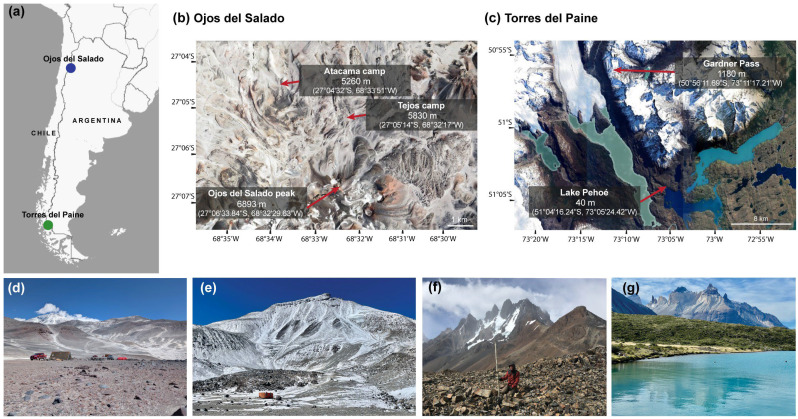
Geographical location of the Ojos del Salado and Torres del Paine sampling sites at (**a**) large scale and (**b**,**c**) local scale. Landscape photograph taken at (**d**) Atacama camp, (**e**) Tejos camp, (**f**) Gardner Pass, and (**g**) Lake Pehoé.

One sampling area was located in the Dry Andes in northern Chile, and the other was in the Chilean Patagonia (Figure 1). Two sites within the Dry Andes were selected for the collection of samples: the Atacama Camp and the Tejos Camp on the Ojos del Salado. The Ojos del Salado region has been the focus of a decade-long research, resulting in a thorough and well-documented characterization of the area [11,22,23,24,25,26,27].

The Atacama camp is located approximately 5.5 km north of the Ojos del Salado massif at an elevation of 5260 m a.s.l, within a flat lava plain. The Tejos site is located approximately 2.5 km north of the Ojos del Salado main volcanic cone, at an elevation of 5830 m a.s.l., in a mountain tundra periglacial environment (Table 1). Surface temperature records at 10 cm depth indicate consistent frost conditions typical of permafrost at the Tejos site, whereas such conditions occur only sporadically at Atacama [25,27]. The active layer is at least >1 m thick at the Atacama, and 70–80 cm at the Tejos camps [26]. Sediments at the Tejos camp are generally coarser than those at Atacama camp. However, the proportion of coarse grains (d > 2 mm) varies with depth, showing the lowest ratios at −35 cm at both the Atacama and the Tejos Camps. At the Atacama site, the bulk density of the regolith was measured 1.3 g/cm^3^ at a depth of 10 cm and 1.5 g/cm^3^ at depths of 35 and 60 cm. The regolith samples at Tejos Camp show particularly low bulk density, with values of 1.1, 1.3, and 1.0 g/cm^3^ at depths of 10, 35, and 60 cm, respectively. At the Atacama site, sediment porosity ranges from 50 to 55% *v*/*v*, and water absorption is high only within the upper 10 cm, dropping sharply at greater depths. At the Tejos site, porosity is even higher, ranging between 55 and 67% *v*/*v* [24].

The Patagonian samples were collected from the Torres del Paine National Park. One sampling site was located in the mountainous Gardner Pass area, at an elevation of 1180 m a.s.l. (Table 1). These samples originate from the tundra, from periglacial conditions, and from an extremely windy location. Control samples were collected from the shore of Lake Pehoé at 40 m a.s.l. and 50 m from the lake. Lake Pehoé is a freshwater lake with an irregular shape and a surface area of 22 km^2^. The main water input to Lake Pehoé is the Paine River, which flows from Lake Nordenskjöld, and it also receives the outflow from the small Lake Sköttsberg [28]. The low-elevation Torres del Paine site was selected as the control for its undisturbed, postglacial environment, which has geomorphological and climatic characteristics comparable to the mountainous sampling area, except for the presence of permafrost.

**Table 1:** Environmental and geographical characteristics of the sampling sites.

**Location**	Ojos del Salado	Ojos del Salado	Torres del Paine	Torres del Paine
**Sampling site**	Atacama Camp	Tejos Camp	Gardner Pass	Lake Pehoé
**Latitude**	27°04′32″ S	27°05′14″ S	50°56′11.69″ S	51°04′16.24″ S
**Longitude**	68°33′51″ W	68°32′17″ W	73°11′17.21″ W	73°05′24.42″ W
**Elevation** (**a.s.l.**)	5260 m	5830 m	1180 m	40 m
**Sampling time**	27 January 2024	29 January 2024	2 March 2024	4 March 2024
**Temperature** (**mean, min, max**) **^1^**	8.8 °C, 3.6 °C, 14.9 °C	3.1 °C, 0.02 °C, 12.8 °C	7.3 °C, 2.9 °C, 12.1 °C	6 °C MAT ^2^ [29]
**Precipitation**	150–200 mm/year [30]		1200 mm/year [31]	
**Sampling depths** (**cm**)	10, 35, 60	10, 35, 60	10, 35	10, 35
**Sample ID**	A24	T24	24TK	24TP
**Description**	Arid, high UV, low bulk density, high porosity		High precipitation, extremely windy	Control samples from the shore of an oligotrophic lake

^1^ Temperature values represent a 7-day interval prior to sampling in case of Ojos del Salado and Gardner Pass sampling sites. ^2^ MAT = mean annual temperature.

### 2.2. Sampling Process, Soil Chemical Analyses

Ojos del Salado samples were collected at the end of January 2024, and the Torres del Paine samples were taken at the beginning of March 2024. In addition to varying geographical parameters such as latitude and altitude, the samples were collected from multiple depths. Although this study was conducted in permafrost environments, all samples were collected from the active layer, the seasonally thawed layer above the frozen ground. Samples of Ojos del Salado were obtained from depths of 10, 35, and 60 cm, while Torres del Paine samples were collected from 10 and 35 cm depths. Two replicate samples were collected at each site and depth. All samples were collected into 50 mL sterile Falcon tubes (Corning, NY, USA) and kept cool until arriving at the laboratory, where they were stored at 10 °C.

Soil samples were air-dried at room temperature before the concentrations of total carbon, nitrogen, and sulfur were determined using a Vario MAX cube CNS analyzer (Elementar, Langenselbold, Germany).

### 2.3. Environmental DNA Extraction and 16S rRNA Amplicon Sequencing

The environmental DNAs from the samples were extracted using a DNeasy PowerSoil Pro Kit (QIAGEN, Hilden, Germany). In consideration of the oligotrophic characteristics of the sampling sites, low DNA concentrations were expected. Therefore, 400–500 mg per sample was used, instead of the recommended 250 mg. Due to the limited number of replicates, parallel DNA extractions and PCR amplifications were performed for each sample to enhance reproducibility. The samples were then pooled prior to sequencing.

The PCR was performed using Bacteria-specific (Bakt_341F: 5′-CCTACGGGNGGCWGCAG-3′ and Bakt_805R: 5′-GACTACNVGGGTATCTAATCC-3′) [32] and Archaea-specific (A519F: 5′-CAGCMGCCGCGGTAA-3′ and Arch855R: 5′-TCCCCCGCCAATTCCTTTAA-3′) [33] primers targeting the V3–V4 variable regions of the 16S rRNA gene. The 5′ end of all primers was elongated with overhang adapters for the Illumina sequencing, provided by SEQme (Dobris, Czech Republic). The adapter sequence for the forward primers was 5′-TCGTCGGCAGCGTCAGATGTGTATAAGAGACAG′ and 5′-GTCTCGTGGGCTCGGAGATGTGTATAAGAGACAG′ for the reverse.

A 20 µL PCR premix was prepared according to the following protocol: 4 µL 5X Phusion HF Buffer, 0.4 µL BSA (20 mg/mL), 0.4 µL 10 mM dNTPs (200 µM each), 0.2 µL 40 µM primers, 0.2 µL Phusion Hot Start II High-Fidelity DNA Polymerase (2 U/µL, Thermo Fisher Scientific, Waltham, MA, USA), 3 or 5 µL DNA template and nuclease free water add to 20 µL. The PCR heat profiles were described by Megyes et al. [34]; the annelation temperature was 55 °C for Bacteria and 60 °C for Archaea. In certain instances, particularly for samples isolated at elevations above 5000 m a.s.l., the number of PCR cycles was increased from 25 to 28. The presence of PCR products was subsequently confirmed through gel electrophoresis using a 1% agarose gel. After pooling the parallels, the concentration of the PCR products was measured using the Qubit assay (Thermo Fisher Scientific, Waltham, MA, USA). Illumina sequencing was carried out by SEQme (Dobris, Czech Republic).

### 2.4. Data Analysis and Statistics

Sequence reads were analyzed in R version 4.3.0 [35] with the DADA2 package 1.28.0 [36] following the instructions on DADA2′s official website (https://benjjneb.github.io/dada2/tutorial.html accessed on 26 October 2025, DADA2 Pipeline Tutorial version 1.16). Taxonomy was assigned to bacterial and archaeal sequence variants using the Silva database version 138.2 [37].

The data analysis was performed using the phyloseq package version 1.44.0 [38]. The phyloseq object was used to integrate Amplicon Sequence Variant (ASV) abundance tables, taxonomic assignments, and metadata into a unified data frame, thereby enabling consistent downstream analyses such as diversity estimation, relative abundance visualization, ordination, and statistics.

Alpha diversity indices were calculated by subsampling the dataset to the smallest library size for Bacteria (*n* = 12,646). The archaeal sequencing of three samples resulted in libraries containing fewer than 1000 reads. Therefore, the alpha diversity for Archaea was determined using relative abundances. Shannon and Inverse Simpson indices were calculated using the estimate_richness function of the phyloseq package version 1.44.0. Statistical differences among sampling places were assessed by one-way analysis of variance (ANOVA) followed by Tukey’s honest significant difference (HSD) post hoc tests using base R functions. The grouping of letters indicating significant differences was generated with the multcompLetters4 function from the multcompView package version 0.1.10 [39]. The visualization was carried out with the ggplot2 package version 4.0.1 [40].

The relative abundance heatmaps and dendrograms were created with the pheatmap package version 1.0.13 [41]. Stacked bar charts were created with dplyr version 1.1.4 [42] and ggplot2 version 4.0.1. Bacterial phyla that were not detected at ≥1% in at least one sample were grouped into the “Others” category. This threshold for bacterial orders was ≥3%, and for Archaea, no threshold was applied.

Beta diversity was assessed based on Bray–Curtis similarity matrices computed with the vegan package version 2.6.10 [43], then non-metric multidimensional scaling (NMDS) was performed to visualize the differences between the samples. Differences in community composition among sampling sites were tested by permutational multivariate analysis of variance (PERMANOVA) using the adonis2 function from the vegan package version 2.6.10. All PERMANOVA tests were performed with 999 permutations.

Differential abundance analysis at the order level was performed using MaAsLin2 (Microbiome Multivariable Associations with Linear Models) version 1.14.1 in R version 4.3.0 [44], with sampling sites as a fixed effect and Control as the reference site. Abundances were normalized with total sum scale (TSS). For Bacteria, only those orders were retained that appeared in ≥1% in at least one sample.

## 3. Results and Discussion

The relative abundance tables, the number of reads, and ASVs derived from next-generation sequencing are provided in Tables S1–S3. The microbial communities were dominated by bacterial taxa in terms of observed diversity, while archaeal taxa were less diverse. Rarefaction curves were sufficient for both prokaryotic groups, even though Archaea had a very low number of reads in some samples (Figure S1).

Although permafrost environments are generally expected to harbor low archaeal biomass [45,46,47,48], the relatively small number of archaeal reads observed in some samples may be influenced by primer bias. In silico evaluations have shown that the commonly used primers (A519F, A855R) incompletely cover certain archaeal phyla. This can lead to the underrepresentation of specific taxa in amplicon libraries [33,49]. Thus, the low archaeal read counts in our study may reflect a combination of genuinely low abundance of these microorganisms in the studied extreme environments and incomplete amplification due to primer bias.

### 3.1. Alpha Diversity of Bacteria

**Figure 2:**
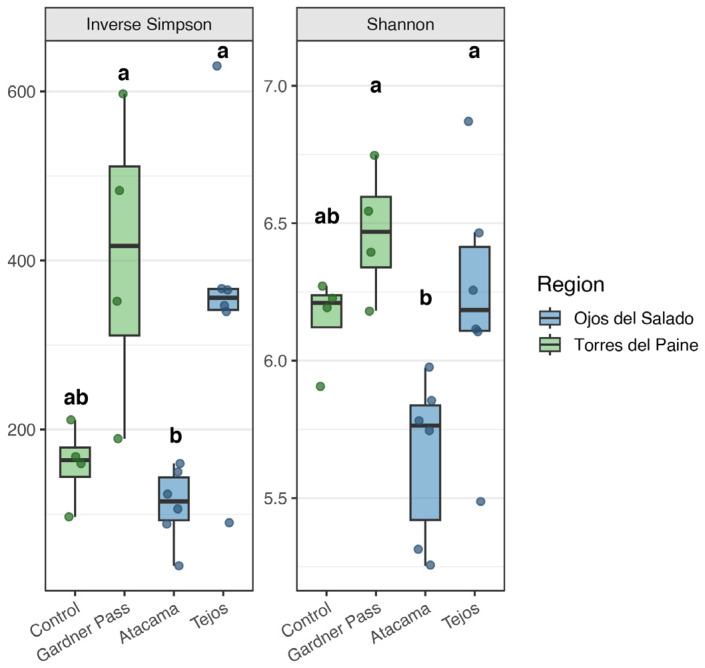
Inverse Simpson and Shannon diversity indices for Bacteria across sampling places. Values were calculated after subsampling to the lowest library size (*n* = 12,646). Letters above each group indicate statistical differences determined by Tukey’s HSD test (groups sharing a letter are not significantly different at *p* < 0.05).

Despite the harsh environmental conditions, a high level of bacterial diversity was observed in the active layer (Figure 2). The highest bacterial alpha diversity was observed in samples from Gardner Pass (Shannon: 6.18–6.75, Inverse Simpson: 189.38–597.06). The elevated alpha diversity observed in this region suggests a substantial capacity for diverse microorganisms to colonize this environment. The region represents wet tundra conditions with high precipitation and intense winds, which may enhance microbial dispersal and broaden the range of taxa in the environment. The control samples were expected to have a higher diversity than the other samples, yet this hypothesis was not supported by the data. It is possible that differences in soil texture, moisture, nutrient content, or microhabitat heterogeneity contributed to these results, although no quantitative measurements are available to support this statement. Despite the inhospitable conditions of Ojos del Salado, its microbial composition does not appear reduced, large number of species can be detected. As previously reported, high alpha diversity is present in the upper layer of permafrost-affected areas. Studies have indicated that the Shannon values from the active layer can reach even higher levels [20,50].

ANOVA performed on the Shannon and the Inverse Simpson diversity values revealed differences between the sampling sites, as indicated by letters in Figure 2. A comparison of sampling locations (Torres del Paine or Ojos del Salado) and depths (10, 35, 60 cm) did not reveal clear differences in the diversity indices.

Despite similar alpha diversity indices between wet and dry regions, wet soils showed a higher number of ASVs (Table S3). This finding may suggest that higher moisture conditions are associated with the presence of low-abundance taxa or habitat specialists within a community, which is consistent with the results of Waqas et al. [51], who examined Alaskan permafrost and reported that the bacterial alpha diversity, as measured by the Observed and Chao1 indices, exhibited a significant increase in wet sites compared to dry sites.

### 3.2. Beta Diversity of Bacteria

**Figure 3:**
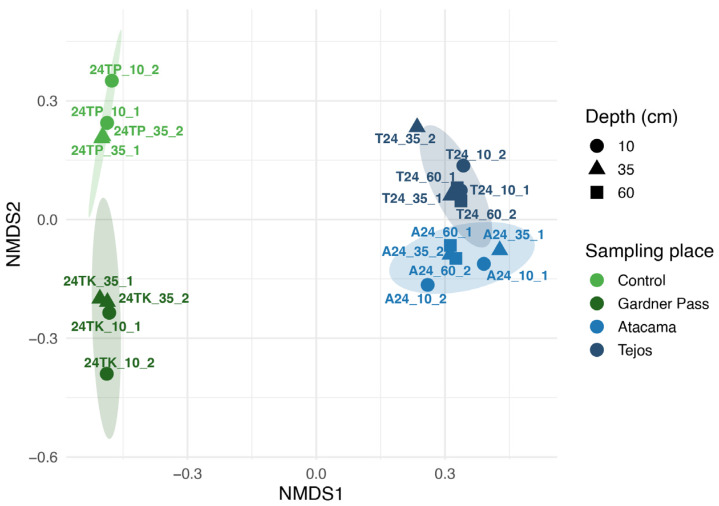
NMDS ordination for Bacteria based on Bray–Curtis distances (stress value = 0.0458).

In the NMDS analysis (stress level = 0.0458), all four sampling sites form a separate group, and samples from the same region are located close to each other (Figure 3). The PERMANOVA results indicate that the sampling sites (Control, Gardner Pass, Atacama or Tejos) and regions (Torres del Paine or Ojos del Salado) were associated with differences in bacterial community composition (*p*-value ≤ 0.001). The sampling site explained a larger proportion of the variance in Bray–Curtis similarities (R^2^ = 0.3819) compared to the sampling region (R^2^ = 0.1678). This pattern suggests that site-specific conditions may play a stronger role in shaping bacterial community structure than broader geographical differences. This finding is consistent with a previous study showing that local factors, like soil moisture content, can outweigh climatic or latitudinal gradients in shaping soil microbial communities [52]. The sampling depth was not associated with detectable differences in bacterial community composition (*p*-value = 0.196).

### 3.3. Relative Abundance of Bacterial Taxa

**Figure 4:**
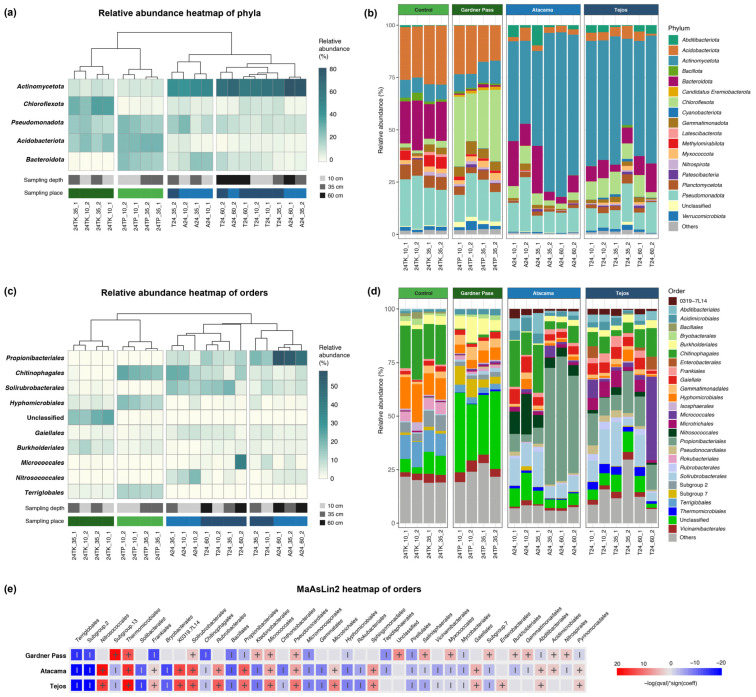
Bacterial community composition at phylum and order taxonomic levels, based on (**a**,**c**) relative abundance heatmaps of most abundant taxa and (**b**,**d**) stacked bar charts. (**e**) Differential abundance analysis of orders across sampling sites using MaAsLin2. The model used Torres del Paine—Control site as a reference. The MaAsLin2 heatmap represents orders that occurred ≥1% of the community in at least one sample.

All together 42 bacterial phyla were identified, where the most abundant were *Actinomycetota* (37.84%), *Pseudomonadota* (14.84%), *Acidobacteriota* (11.51%), *Chloroflexota* (10.23%), and *Bacteroidota* (10.12%). These phyla are generally reported to be dominant in both high-altitude [18] and tundra soils [50]. Less than 1% of the ASVs were categorized as unclassified phyla (Figure 4a,b).

The Ojos del Salado and Patagonian samples were divided into two groups by the Bray–Curtis dendrograms (Figure 4a,c). The environments of the Atacama and Tejos camps were highly similar, which may provide an explanation for the inaccurate differentiation between the two groups in the dendrograms.

In addition to the presence of permafrost, water availability is a key environmental factor that shapes a microbial community [51]. This study examined samples from the arid regions of the Andes, which were dominated by *Actinomycetota*, a phylum characterized by its high resistance to drought, and have been isolated from the hyperarid-arid margin of the Atacama Desert [53], high-altitude desert soils in the Central Andes exposed to extreme UV radiation [54]. Similarly, in the Barrancas Blancas plain (Puna de Atacama), the driest reference site, located furthest from the temporary lake and under reduced hydrological influence, was dominated by *Actinomycetota* [55], in agreement with our observation that this phylum is associated with drier Andean environments. A comparative study of global active layer microbiomes revealed that the relative abundance of *Actinomycetota* was high in all samples [48]. Members of the *Actinomycetota* can form spores when water decreases in the environment, while others change their cytoplasm structure and cell wall thickness in order to form cyst-like cells, which are more resistant to unfavorable environmental conditions [56,57]. The presence of liquid water and even atmospheric humidity can determine whether cells remain metabolically active or enter dormant states. As water activity declines to approximately 0.88 aw, even highly desiccation-tolerant bacteria may enter a state in which metabolic processes halt, although the cells remain viable [56]. Since DNA is prone to degradation, the long-term survival strategy is more likely that cells remain metabolically active and repair their DNA than to enter dormancy [58]. The large number of *Actinomycetota* in the Ojos del Salado samples may suggest that at the time of sampling, the environments were arid and unfavorable, since the presence of *Actinomycetota* increases with water scarcity [21,51].

From the *Actinomycetota* phylum, the most abundant orders were *Propionibacteriales* and *Solirubrobacterales* in the Ojos del Salado samples (Figure 4c,d). Almost all members of the *Propionibacteriales* order belonged to the *Nocardioidaceae* family (99.8%), and 94.69% of them were classified into the *Nocardioides* genus (Table S1). *Nocardioides* can endure various oligotrophic conditions and are even able to break down several pollutants that are hard to degrade [59], giving them an advantage in low-nutrient environments such as the Ojos del Salado (Table S4). Members of the order *Solirubrobacterales*, particularly the genus *Solirubrobacter,* have been isolated from arid and high UV-exposed environments [60]. In addition to Unclassified genera, *Solirubrobacter* was found in high abundance in all Ojos del Salado samples (Table S1).

*Chloroflexota* had a high abundance in the Gardner Pass samples, accounting for 22.82–34.27% of the bacterial community in these four samples. The aquatic systems of Torres del Paine are distinguished by their low temperatures, limited nutrients, and low dissolved organic carbon (DOC) concentrations [61]. The studied region receives approximately 1200 mm of precipitation annually [31]. Previous studies found that the abundance of *Chloroflexota* in permafrost is associated with relatively lower nutrient availability and higher moisture content [20], and they revealed that *Chloroflexota* was the dominant phylum in alpine, oligotrophic active layer with mean annual precipitation of 941 mm [45]. In line with these observations, *Chloroflexota* showed the highest relative abundance in the wet region of the Andes. Similarly, *Bacteroidota* has been reported to have a significant positive correlation with mean annual precipitation [52]. It showed the highest abundance in control samples taken from the shore of an oligotrophic lake in Patagonia.

Our findings indicate that phototrophic taxa did not appear, or their appearance was minimal. Although photosynthetic microorganisms have been isolated from permafrost [62], and high altitude does not exclude the presence of *Cyanobacteria*, as they were reported from 5900 m a.s.l., where liquid water was present [21]. In that prior study, *Cyanobacteria* could not be detected directly on the surface due to high UV radiation, but near the surface or in shaded areas [21]. Samples from the Ojos del Salado analyzed in this study showed the presence of phyla that indicate aridity, which is unfavorable for *Cyanobacteria* due to their requirement for high water activity (ranges from 1 to 0.6) [63]. The environmental conditions in Torres del Paine do not prevent the emergence of *Cyanobacteria*, but they cannot be detected. It is possible that the presence of these taxa may be suppressed by other taxa, or they might occur closer to the surface than 10 cm. In such extreme environments, suitable niches for *Cyanobacteria* likely require protection from UV radiation and sufficient light. In this study, however, all samples were collected from depths of at least 10 cm, where light penetration was presumably insufficient to support photosynthesis.

Differential abundance analysis using MaAsLin2 identified multiple bacterial orders significantly associated with the sampling sites (Figure 4e). *Terriglobales* and *Solibacterales* were depleted at Atacama and Tejos, while *Nitrosococcales* and *Thermomicrobiales* were enriched at these sites. In accordance with a previous report on hyper-arid Andean environments [64], we identified taxa potentially involved in initial soil formation processes. Members of *Frankiales* were more abundant in the extreme sites of Ojos del Salado, while *Burkholderiales* were primarily associated with the Gardner Pass samples, suggesting a shift in community composition along the environmental gradient.

### 3.4. Diversity of Archaea

**Figure 5:**
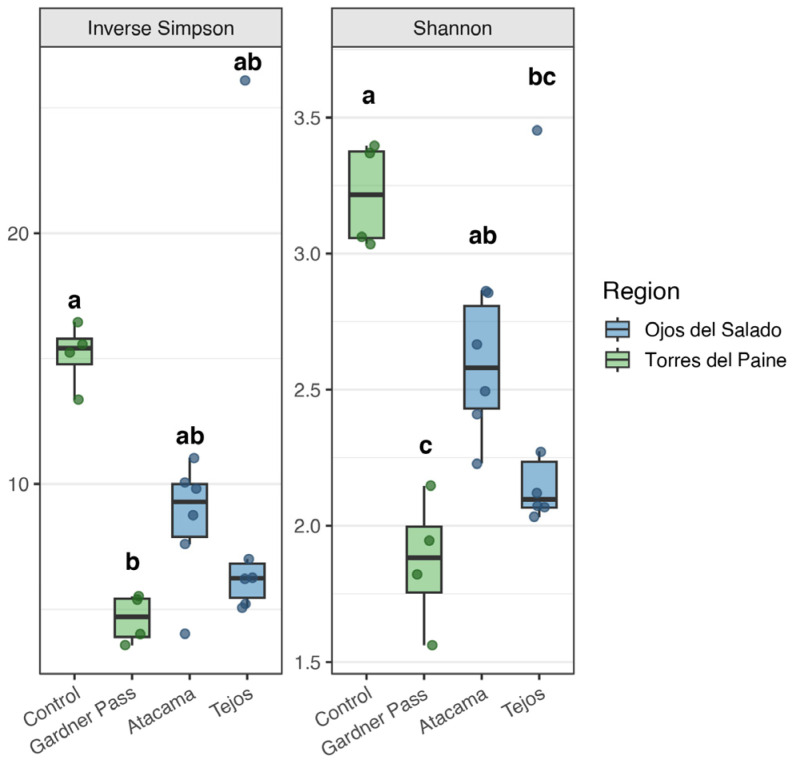
Inverse Simpson and Shannon diversity indices for Archaea across sampling places. Values were normalized by total sum scaling (TSS). Letters above each group indicate statistical differences determined by Tukey’s HSD test (groups sharing a letter are not significantly different at *p* < 0.05).

The archaeal pattern of the Ojos del Salado and the Torres del Paine sample from the mountainous region showed differences compared to the control samples, as shown by Tukey’s HSD tests in Figure 5.

**Figure 6:**
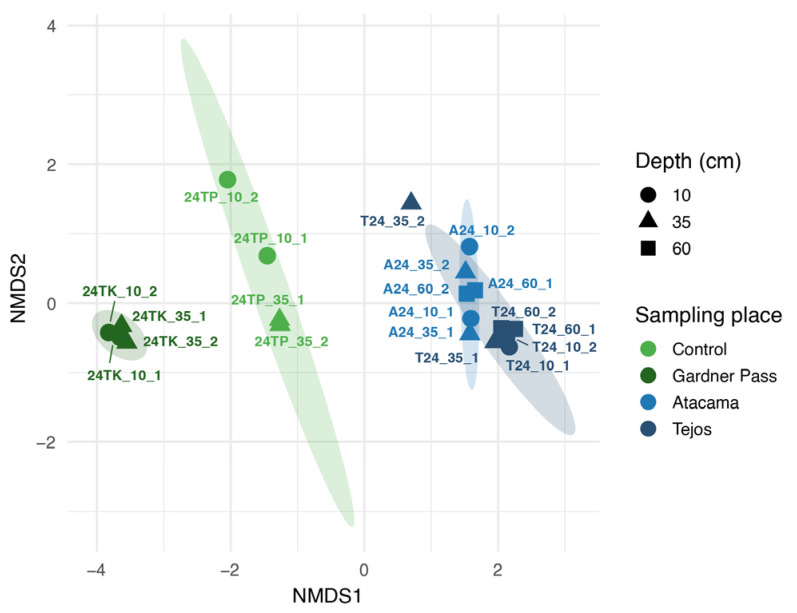
NMDS ordination for Archaea based on Bray–Curtis distances (stress value = 0.0177).

The NMDS analysis (Figure 6) and the PERMANOVA analysis for Archaea showed patterns similar to the bacterial results. The impact of the sampling depth was found to be less considerable than that of the bacterial (*p*-value = 0.28), while the sampling place (*p*-value ≤ 0.001, R^2^ = 0.6388) and sampling region (*p*-value ≤ 0.001, R^2^ = 0.3714) were associated with differences in archaeal community composition.

**Figure 7:**
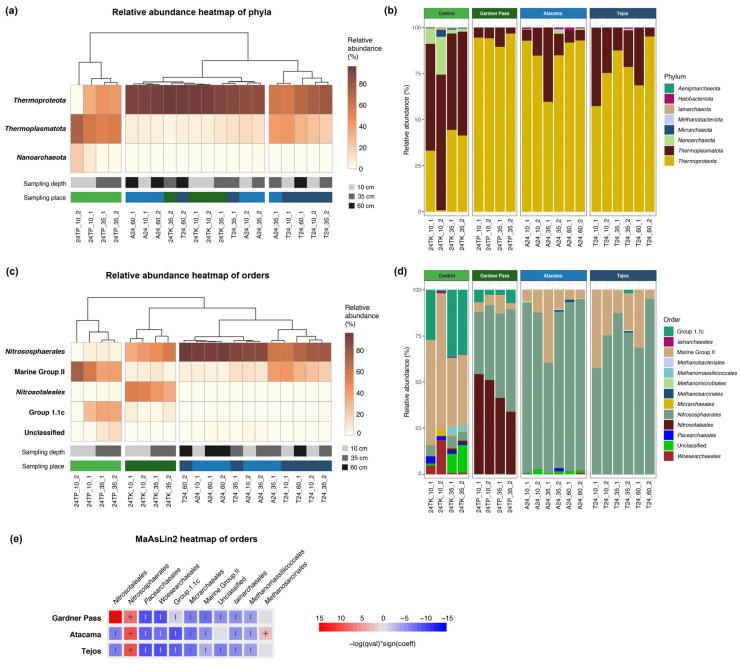
Archaeal community composition at phylum and order taxonomic levels, based on (**a**,**c**) relative abundance heatmaps of most abundant taxa and (**b**,**d**) stacked bar charts. (**e**) Differential abundance analysis of orders across sampling sites using MaAsLin2. The model used Torres del Paine—Control site as a reference.

Although the control samples had a higher alpha diversity than the rest of the samples, many ASVs were assigned to unclassified or non-formal archaeal clades such as Marine Group II and Group 1.1c (Figure 7). The differential abundance analysis of archaeal communities (Figure 7e) revealed that members of *Thermoproteota* (previously *Thaumarchaeota*), particularly *Nitrososphaerales*, were enriched at permafrost-related sampling sites, and *Nitrosotaleales* was most abundant at Gardner Pass. These orders are known as ammonia-oxidizing archaea (AOA). They are autotrophic and able to fix their own carbon from inorganic CO_2_. Ammonia oxidizers are regarded as specialist microorganisms, as they typically rely on ammonia as the sole source of reducing power. While AOAs are generally adapted to low ammonia concentrations, it is also important to acknowledge that ammonia oxidizers have a wide range of ammonia affinities. Since nitrifiers in the environment are frequently exposed to low and fluctuating substrate concentrations, the affinity for ammonia is likely an important survival factor [65]. Our results indicated that AOA were present in high abundance in permafrost-related environments and were scarce in the control samples.

Although ammonia-oxidizing bacteria (AOB) like *Nitrosococcales* are present in these environments (Figure 4c,d), the dominance of ammonia-oxidizing Archaea suggests that nitrification in these samples is predominantly driven by Archaea. A previous study found that AOB only grew at high ammonia concentrations, while AOA favored low, intermediate, and high ammonia concentrations too [66]. The samples analyzed in this study are characterized as oligotrophic, indicating the presence of AOA rather than AOB (Table S4).

Our research suggests that phototrophic or anaerobic taxa did not appear, or only appeared to a negligible extent, among Bacteria and Archaea. The absence of anaerobic microorganisms is also not evident, 10–15% of anaerobic microorganisms can live in different global permafrost environments [48]. This is likely due to the coarse nature of the analyzed active layer and the strong winds that restricted the presence of anaerobes.

The community composition suggests the presence of chemolithotrophic metabolic strategies, which are advantageous in carbon-limited environments where inorganic electron donors and carbon sources are more consistently available than organic substrates. Waldrop et al. proposed that adaptations to energy acquisition and substrate availability are among the strongest selective pressures shaping permafrost microbial communities [46].

## 4. Conclusions

The comparison of prokaryotic taxonomic diversity in the largely unexplored permafrost regions located in different climatic zones (dry and wet) of the Chilean Andes revealed that these environments are inhabited by diverse and distinct microbial communities, characterized by a higher representation of Bacteria rather than Archaea. The structure of bacterial communities may be related to the water activity of the permafrost active layer. The moist Patagonian samples showed higher biodiversity, with larger abundances of *Chloroflexota* and *Bacteroidota*, while the hyper-arid Ojos del Salado samples were dominated by *Actinomycetota*, consistent with extreme drought stress. Archaeal communities were less diverse but exhibited strong functional specialization. The high abundance of ammonia-oxidizing taxa suggests that nitrification is predominantly driven by Archaea in these extreme environments. These findings support our initial hypothesis that geographic and climatic differences shape microbial community composition. However, contrary to our expectations, sampling depth did not have a detectable influence on community structure. The absence of a measurable depth-related effect in this dataset indicates that vertical variations were relatively weak compared to site-specific environmental differences.

## Data Availability

Raw sequencing data have been deposited in the Sequence Reads Archive under the BioProject accession number: PRJNA1394536.

## Supplementary Materials

The following supporting information can be downloaded at https://www.mdpi.com/article/10.3390/microorganisms14030613/s1. Figure S1: Rarefaction curve for Bacteria and Archaea; Table S1: Relative abundance table for Bacteria; Table S2: Relative abundance table for Archaea; Table S3: Number of reads and ASVs of the samples; Table S4: Soil chemical analyses (C, N, S) of Ojos del Salado samples.
