# Supplementary figures and images for: The Taxonomic Diversity of Prokaryotic Communities from Permafrost Active Layers of the Chilean Andes
Source: Microorganisms. 2026 Mar 9;14(3):613. doi: 10.3390/microorganisms14030613 (PMC13028659; doi:10.3390/microorganisms14030613)

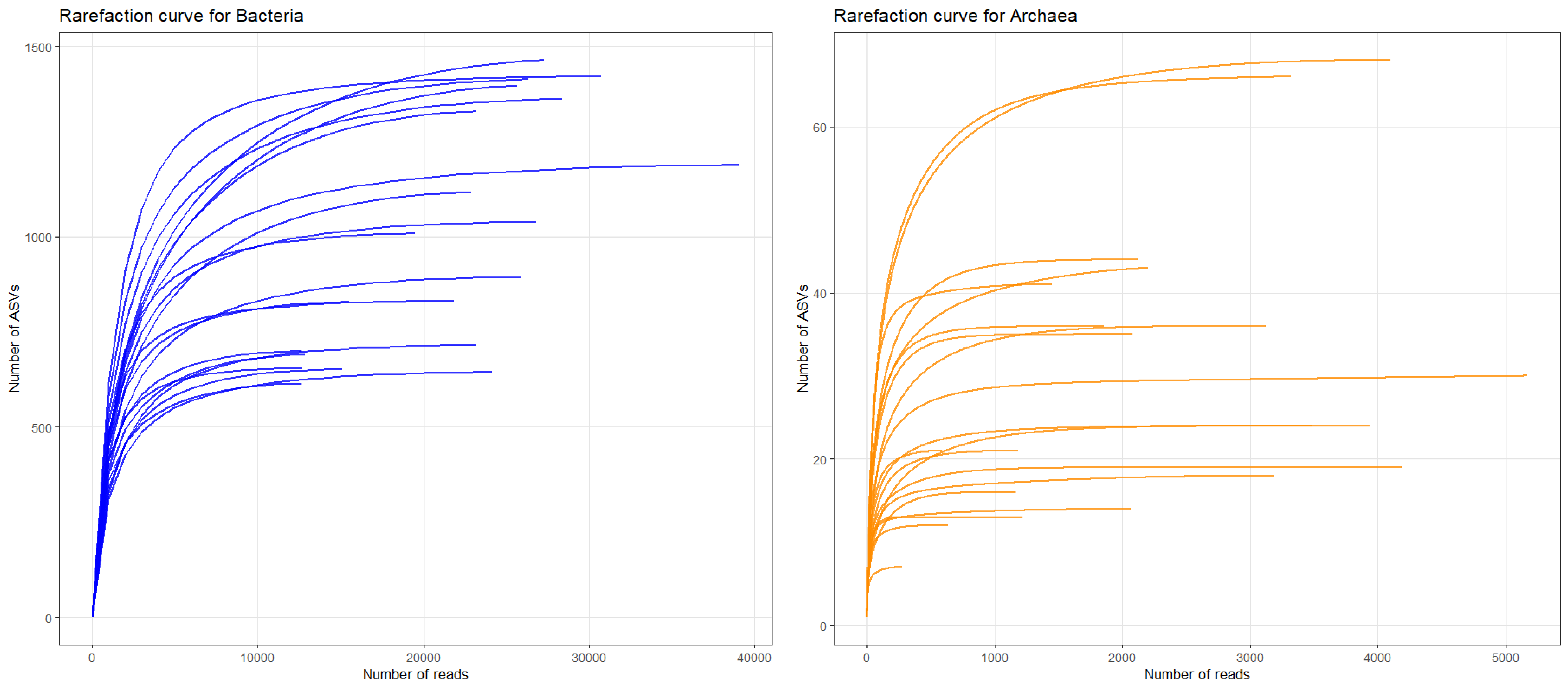

Supplement: Supplementary file 1 [file microorganisms-14-00613-s001.zip › microorganisms-4096875-supplementary/Supplementary Figure S1.png]
